# Supplementary material for: Differential equation-based minimal model describing metabolic oscillations in Bacillus subtilis biofilms
Source: R Soc Open Sci. 2020 Feb 5;7(2):190810. doi: 10.1098/rsos.190810 (PMC7062081; doi:10.1098/rsos.190810)
Supplement: Supplements [file rsos190810supp1.docx]

**SUPPLEMENT TO**

**Differential equation based minimal model describing metabolic oscillations in *Bacillus subtilis* biofilms**

Ravindra Garde^1,2^, Bashar Ibrahim^1,4,5^, Ákos T. Kovács^3^, Stefan Schuster^1*^

^1^Dept. of Bioinformatics, Matthias Schleiden Institute, Friedrich Schiller University Jena,

Ernst-Abbe-Platz 2, 07743 Jena, Germany

^2^Max Planck Institute for Chemical Ecology Hans-Knöll-Str. 8, 07745, Jena, Germany

^3^Bacterial Interactions and Evolution Group, DTU Bioengineering, Technical University of Denmark, Søltofts Plads Building 221, 2800 Kgs. Lyngby, Denmark

^4^Centre for Applied Mathematics and Bioinformatics, Gulf University for Science and Technology, Hawally 32093, Kuwait

^5^Department of Mathematics and Natural Sciences, Gulf University for Science and Technology, Hawally 32093, Kuwait

*Corresponding Author

**Applying the quasi-steady-state approximation to ammonia**

Just as in the case of the QSSA variable *G_i_* (see main text for details), we apply the same approximation to the variable *A*.

$A=\frac{k_{5}}{k_{3}} G_{i}$ (S1)

From Eq. (1), we derive a reduced system:

$\frac{{dG}_{p}}{dt}{=k}_{1}G_{E}G_{p}-k_{4}G_{p}-{\frac{{k_{2}k}_{5}}{k_{3}} G_{i}G}_{p}$ (S2)

$\frac{dG_{i}}{dt}=k_{4}G_{p} - k_{5}G_{i}$ (S3)

The system shows two steady states:

${G_{p}}_{1}={G_{i}}_{1}=0$ (S4a,b)

${G_{p}}_{2}=\frac{(k_{1}G_{E}-k_{4})k_{3}}{k_{2}k_{4}}$ , ${G_{i}}_{2}=\frac{(k_{1}G_{E}-k_{4})k_{3}}{k_{2}k_{5}}$ (S5a,b)

(see Eqs. (3a,c)). The Jacobian matrix reads:

$\mathbf{M}=\left( \begin{matrix} k_{1}G_{E}-k_{4}-\frac{k_{2}k_{5}}{k_{3}}G_{i} & -\frac{k_{2}k_{5}}{k_{3}}G_{p} \\ k_{4} & -k_{5} \end{matrix} \right)$ (S6)

For the trivial steady state (TSS, Eq. (S4)), it leads to:

$\mathbf{M}=\left( \begin{matrix} k_{1}G_{E}-k_{4} & 0 \\ k_{4} & -k_{5} \end{matrix} \right)$ (S7)

For matrices with such a triangular structure, the eigenvalues are given by the diagonal elements. In our case:

$\lambda_{1} = k_{1}G_{E}-k_{4}$, $\lambda_{2}= -k_{5}$ (S8)

In any case, the eigenvalues are real, so that not even damped oscillations are possible. For *k_1_G_E_* *< k_4_*, both eigenvalues are negative, so that the trivial steady state is a stable node. For *k_1_G_E_ > k_4_*, one eigenvalue is negative and the other one positive. The steady state then is unstable, it is a saddle point.

For the NTSS (Eq. (S5)), the Jacobian matrix becomes:

$\mathbf{M}=\left( \begin{matrix} 0 & -\frac{k_{1}G_{E}-k_{4}}{k_{4}}k_{5} \\ k_{4} & -k_{3} \end{matrix} \right)$ (S9)

The characteristic equation reads:

$\lambda^{2}+k_{3}\lambda+\left( k_{1}G_{E}-k_{4} \right)k_{5}=0$ (S10)

This has the solutions

$\lambda_{1/2}= -\frac{k_{3}}{2}\pm\sqrt{\frac{k_{3}^{2}}{4}-\left( k_{1}G_{E}-k_{4} \right)k_{5}}$ (S11)

Now, we distinguish three cases:

1. For $k_{1}G_{E}$ < *k*_4_, the term under the square root is positive, so that the root is real. Moreover, it is larger than *k*_3_/2. Thus, one eigenvalue is negative and the other one positive. The steady state then is unstable, it is a saddle point.
2. For 0 < $k_{1}G_{E}$ – *k*_4_ < $\frac{k_{3}^{2}}{4k_{5}}$, the root is again real. It is less than *k*_3_/2, though. Both eigenvalues are negative; the steady state is a stable node.
3. For $k_{1}G_{E}$ – *k*_4_ > $\frac{k_{3}^{2}}{4k_{5}}$, the root is imaginary. Both eigenvalues are complex numbers, with the same negative real part −*k*_3_/2. The steady state is a stable focus. This state is, thus, reached by damped oscillations.

The transition between stable node and stable focus occurs at $k_{1}G_{E}$ – *k*_4_ = $\frac{k_{3}^{2}}{4k_{5}}$. The results of this approximation are similar to those in the main text section on QSSA. We can conclude that the limit-cycle oscillations vanish if ammonia diffuses very fast.
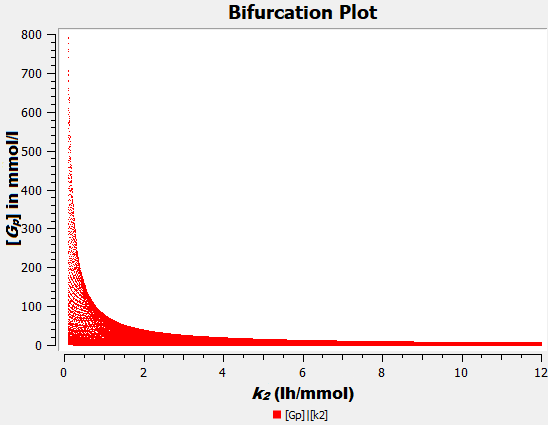
**Figure S1:** Bifurcation plot of *G_p_* versus *k_2_*. It can be seen that the model is not very sensitive to *k_2_* in terms of amplitude of oscillations if *k_2_* ≥ 2 h^-1^.


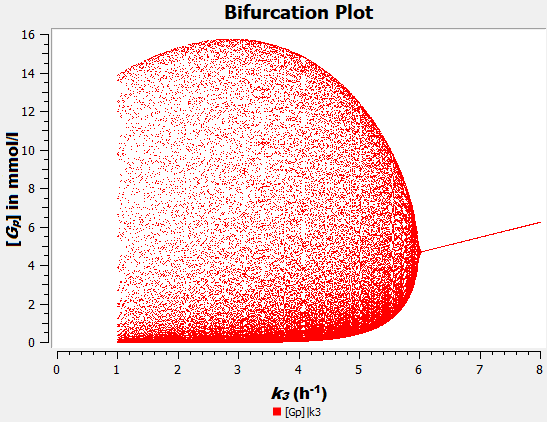
**Figure S2**: Bifurcation plot of *G_p_* versus *k_3_*. The model is sensitive to *k_3_* if *k_3_* ≥ 4 h^-1^ in terms of amplitude of oscillations.


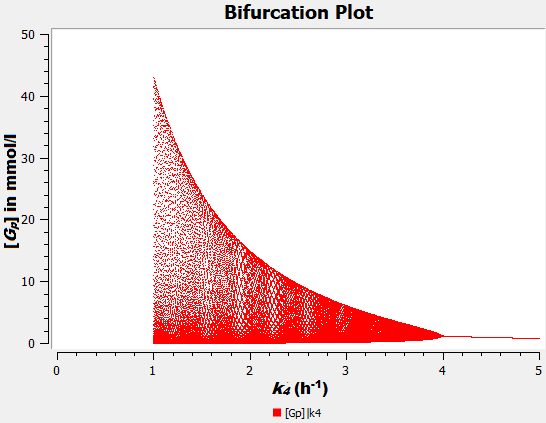
 **Figure S3**: Bifurcation plot of *G_p_* versus *k_4_*. The model is sensitive to *k_4_* if *k_4_* ≤ 3 h^-1^ in terms of amplitude of oscillations.


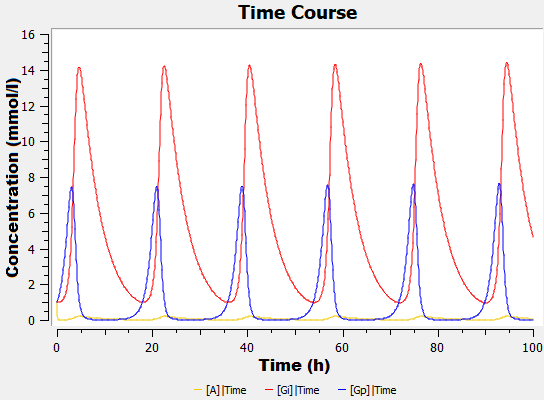
**Figure S4:** Time course after introducing self-amplification to *G_i_* in addition to *G_p_*. This can be obtained by changing the term *k_4_G_p_* to *k_4_G_i_G_p_* in eq (1). The oscillations become spike-like but no dramatic difference to the results of the minimal model can be seen. In order to obtain realistic oscillations, the parameter values needed to be changed. The parameters from Table 1 were reduced to 10% while *k_3_* was increased to 15.93 h^-1^. In the main text, we only use self-amplification for *G_p_*.


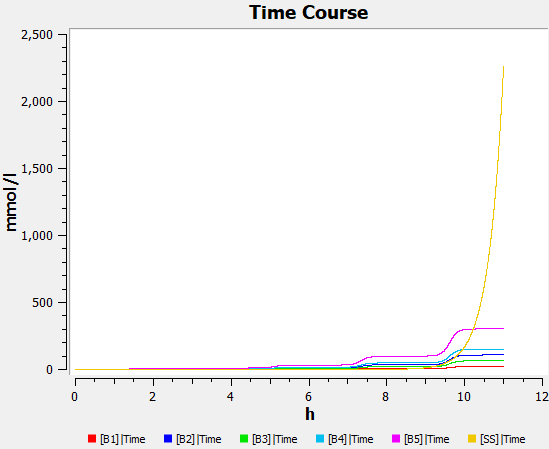


**Figure S5:** Plot of the time course of growth as calculated from Eq. (4) for various initial values of *G_p_* from 1 mmol/l – 10 mmol/l with a step size of 1 mmol/l (all wavy curves). On average the curves have a doubling time of about 99 minutes. The black monotonic curve (initial value: 10^-9^ mmol/l) indicates the growth calculated by the steady state values. It can be seen that the steady state growth rate overtakes the oscillating growth rate at about 10.5 hours.

**Table S1**: Sensitivity analysis for all variables at steady state with respect to all parameters. Darker shades denote higher sensitivity. Positive sensitivity is shown in green and negative in red. White means that the variable is not sensitive to that parameter.

**a:** Values indicate unscaled derivatives at steady state.

|  | *k_1_* | *k_2_* | *k_3_* | *k_4_* | *k_5_* |
| --- | --- | --- | --- | --- | --- |
| [*A*] | 5.6604 | -0.2944 | 0 | -0.1887 | 0 |
| [*G_i_*] | 9.8441 | -0.5120 | 0.6790 | -0.3281 | -1.1798 |
| [*G_p_*] | 11.3208 | -0.5888 | 0.7809 | -1.9392 | 0 |

**b**: Values indicate scaled derivatives at steady state.

|  | *k_1_* | *k_2_* | *k_3_* | *k_4_* | *k_5_* |
| --- | --- | --- | --- | --- | --- |
| [*A*] | 1.2416 | -1 | 0 | -0.2416 | 0 |
| [*G_i_*] | 1.2416 | -1 | 1 | -0.2416 | -1 |
| [*G_p_*] | 1.2416 | -1 | 1 | -1.2416 | 0 |
